# Differences in the count of blood cells pre-and post-chemotherapy in patients with cancer: a retrospective study (2022)

**DOI:** 10.3389/fmed.2025.1485676

**Published:** 2025-04-16

**Authors:** Tadele Derbew Kassie, Bayu Wondimneh Yimenu, Gelagey Baye Temesgen, Rahel Asres Shimelash, Aysheshim Asnake Abneh

**Affiliations:** ^1^Department of Public Health, College of Health Science, Debre Markos University, Debre Markos, Ethiopia; ^2^Biomedical Department, College of Medicine, Debre Markos University, Debre Markos, Ethiopia; ^3^Department of Nursing, College of Health Science, Debre Markos University, Debre Markos, Ethiopia

**Keywords:** blood cell type, chemotherapy, differences, patient with cancer, northern Ethiopia

## Abstract

**Introduction:**

Cancer is a disease characterized by uncontrolled cell growth that can invade and spread to other body parts. Drugs used for chemotherapy can cause damage to non-cancer cells and lead to a low blood cell count. There are controversial findings regarding the differences in the counts of blood cell types. Studies on the counts of blood cell types before and after chemotherapy are limited. Therefore, this study aimed to address this gap.

**Methods:**

A retrospective study was conducted on 354 patients from 1 September 2022 to 1 October 2022 to compare blood cell type profiles in pre-and post-operative periods. A chi-squared test and paired *t*-test were performed to analyze the data.

**Results:**

Data were collected from 354 patients. The mean age of the respondents was 41.26 (±16.67) years. At the initial diagnosis, 167 patients (47%) were categorized as having stage III cancer, while 159 patients were categorized as having stage IV cancer. Before and after chemotherapy the mean heamoglobin level is 12.95 g/dL and 12.30 g/ dL respectively and the prevalence of anemia among cancer patients before chemotherapy and after chemotherapy was 25.14% (95%:20.88, 29.94) and 35.54% (95%: 30.75; 40.73), respectively. The mean value of neutrophils before and after chemotherapy was 52.72 and 50.82%, respectively. The frequency of neutropenia among cancer patients was 22.32% (95% 18.26; 26.96) before chemotherapy and 27.97% (95%:23.52; 32.88) after chemotherapy, and the mean value of lymphocytes before and after chemotherapy was 37.50 and 34.29%, respectively. Lymphopenia among the study participants was 16.38% (95%:12.87; 20.62) before chemotherapy and 17.51% (95%13.88, 21.84) after chemotherapy. Among patients with stomach, rectal, and bone cancers, there was no significant difference in the counts of all blood cell types before and after chemotherapy. The mean reduction in platelets was 23. 51 × 10^3^cells/mm^3^ (*p* = 0.001). For red blood cells (RBCs), the mean decrease was 0.63 × 10^6^cells/mm^3^ (*p* = 0.08), and for white blood cells (WBCs), it was 2.49 × 10^6^ cells/mm^3^ (*p* = 0.012).

**Conclusion and recommendation:**

After chemotherapy, all hematological parameters showed a decrease, indicating that chemotherapy significant impacted the levels of these hematological parameters. Therefore, replacement therapy should be considered after chemotherapy.

## 1 Introduction

Cancer is a disease characterized by uncontrollable cell growth that can invade and spread to other parts of the body. There are numerous potential causes for cancer, including an individual’s genetic, environmental, or constitutional traits (4). Cancer is considered one of the most serious and deadly illnesses of the 21st century (5). The type and location of the cancer, the conventional medical procedures and treatment protocols prevalent in a patient’s culture, and the cancer stage at the time of initial diagnosis influence the treatment a patient receives. Cancer treatment might involve various approaches, including radiation therapy, chemotherapy, and surgery. However, patients are at risk for several adverse effects, and these treatments are typically not curative. As a result, cancer treatments often has many side effects, including anemia, infection, bleeding issues, nausea, vomiting, allergic reactions, pain, soreness, constipation or diarrhea, hair loss, sore mouth, increased energy, and trouble falling asleep (6). Hematological abnormalities are dangerous side effects of chemotherapy. This disease affects the blood and its components, such as red blood cells (RBCs), white blood cells (WBCs), platelets, hemoglobin, and plasma. Hematological abnormalities, particularly anemia, are the most prevalent complications among patients undergoing chemotherapy (7). These conditions can lead to significant health issues, including fatigue, increased infection risk, clotting disorders, and severe bleeding tendencies (8).

According to a study conducted on the effect of chemotherapy and radiotherapy on red blood cells and hemoglobin in cancer patients, there was a difference in the level of red blood cells pre-chemotherapy and post-chemotherapy (9). Administration of drugs for chemotherapy causes damage to non-cancer cells, and this leads to a low count of white blood cells following chemotherapy (10).

Chemotherapy affects the counts of blood cells and biochemical profiles in different ways. It functions as an alkalizing agent and causes the bone marrow’s hematopoietic stem cells to disappear gradually (11). Chemotherapy usually works by blocking the creation of deoxyribonucleic acid (DNA), proteins, and microtubules, which results in the death of cells or the prevention of their proliferation (12). Chemotherapeutic drugs cause (13) deoxyribonucleic acid damage during replication by covalently binding to the deoxyribonucleic acid of bone marrow cells, generating intra-and inter-strand cross-links. Platelets, leukocytes, and hemoglobin levels all drop. A study conducted on the effect of chemotherapy on hemoglobin revealed that there was no significant association between hemoglobin and chemotherapy (14).

Chemotherapy-treated cancer patients have been shown to experience immune system changes and anemia. Frequent, intense chemotherapy has been shown to decrease immune cells and lead to opportunistic infections (15). Chemotherapy side effects are noticeable in the bone marrow, a key source of pluripotent hematopoietic stem cells (16).

Moreover, the use of drugs that are specifically designed to be cytotoxic in the complicated field of cancer treatment invariably has detrimental effects on the blood cell profile. Many of these medications are mostly metabolized in the liver; therefore, while administering chemotherapy, liver-drug interactions need to be taken into consideration (17). Chemotherapy administration poses a challenge to the careful regulation and equilibrium of liver function. Due to the liver’s easy absorption of the majority of chemotherapy medicines, up to 85% of patients experience liver stenosis (17). Chemotherapy administered repeatedly can cause permanent damage to the liver by drawing in inflammatory cells and changing the levels of enzymes, such as lactate dehydrogenase (LDH), alanine aminotransferase (ALT), aspartate aminotransferase (AST), and alkaline phosphatase (ALP), and it can lead to a decrease in erythropoietin (18).

The volume of platelet counts after chemotherapy in patients with cancer showed a decrement due to the effect of chemotherapy. However, a study conducted on ovarian cancer showed that there was an increase in thrombocytes (19).

There are controversial findings regarding the differences in the counts of blood cell types across various studies. Most studies have focused on the relationship between specific cancer types and individual blood cell types. There are limited studies on the counts of blood cell types before and after chemotherapy in patients with different cancer conditions in the specified areas. In addition, there are few studies on the abnormalities of hematological parameters in these patients. Therefore, assessing these abnormalities has profound implications for both public health and clinical practice. The findings of this study will support the diagnosis, management, and prevention of complications. Additionally, they will aid in resource allocation, health policy development, and epidemiological surveillance. Understanding the changes in hematological parameters is substantial for implementing appropriate interventions. Therefore, this study aimed to address this gap and provide different scientific bases for various stakeholders.

## 2 Methods

This study was conducted at Mekele Comprehensive Specialized Hospital in the northern part of Ethiopia. The hospital has two oncology centers: pediatric oncology center and adult oncology center.

### 2.1 Study design

To compare hematological parameters in pre-and post-chemotherapy cancer patients, an institutional-based retrospective study design was employed from 1 September 2022 to 1 October 2022.

### 2.2 Populations

All patients with various types of cancer who attended the hospital for chemotherapy were the source population, and patients who started chemotherapy treatment between September 2018 and September 2022 were the study population.

### 2.3 Inclusion and exclusion criteria

#### 2.3.1 Inclusion criteria

All cancer patients who had undergone chemotherapy and had complete medical records, including demographic information, cancer stage, and various types of blood cell counts, were included in the study.

#### 2.3.2 Exclusion criteria

- Patients who underwent surgery, radiotherapy, or immunotherapy were excluded from the study
- Patients with comorbid conditions, such as diabetes mellitus, chronic obstructive pulmonary disease, tuberculosis, HIV/AIDS, chronic kidney disease, congestive heart failure, inflammatory bowel diseases, and viral hepatitis, were excluded as these diseases compromise hematological parameters.
- Patients who were lost to follow-up before completing the IV cycle of the treatment were excluded from the study

### 2.4 Sampling method and sample size determination

All samples that met the inclusion criteria were included. The total sample size was 354 patients. The required data were selected from these 354 patients, who were admitted to the oncology unit between September 2018 and September 2022.

### 2.5 Operational terms

Pre-chemotherapy levels of blood cell types: These were the levels of blood cells at the time of diagnosis and before starting chemotherapy.

Post-chemotherapy levels of blood cell types: These were the levels of blood cells after completing the phase IV treatment of chemotherapy.

Hemoglobin level: The hemoglobin level in female patients was considered low if below 12 g/dL, normal if between 12 g/dL and 16 g/dL, and high if above 16 g/dL. In male patients, it was categorized as low if below 13 g/dL, normal if between 13 g/dL and 18 g/dL, and high if above 18 g/dL **(17).**

### 2.6 Variables of the study

#### 2.6.1 Independent variable

Chemotherapy was the independent variable.

#### 2.6.2 Dependent variables

Hematological parameters such as red blood cells, hemoglobin, hematocrits, white blood cells, neutrophils, lymphocytes, and platelets were the dependent variables.

#### 2.6.3 Data collection tools

A data extraction method was used to collect the required information from secondary data sources. Therefore, to collect the necessary information, the register logbook was first reviewed, and the patients’ medical registration numbers were recorded. Redundant medical registration numbers were removed using Excel. Finally, data were collected from SmartCare and individual patient folders.

### 2.7 Method of data processing and analysis

First, the data were entered and cleaned using EPIDATA (version 4.6). Next, the data were exported to Statistical Package for Social Sciences (SPSS) (version 23) for analysis. Continuous data were analyzed using means, and categorical data were analyzed using Pearson’s chi-squared test to determine the presence of categorical differences before and after chemotherapy. The changes in hematological parameters before and after chemotherapy were compared using a paired *t*-test. All assumptions of the test were met.

### 2.8 Data quality control

The data preparation checklist was adapted from different articles. The prepared checklist format was first tested on 50 samples before starting the actual data collection. The purpose of the study was briefly explained to the data collectors. The data collectors included one nurse from the oncology unit, one SmartCare administrator, and one card room worker. A one-day training session was attended by these data collectors. Daily supervision was conducted to ensure proper completion of the checklist.

## 3 Results

### 3.1 Sociodemographic characteristics of the patients

**Figure 1:**
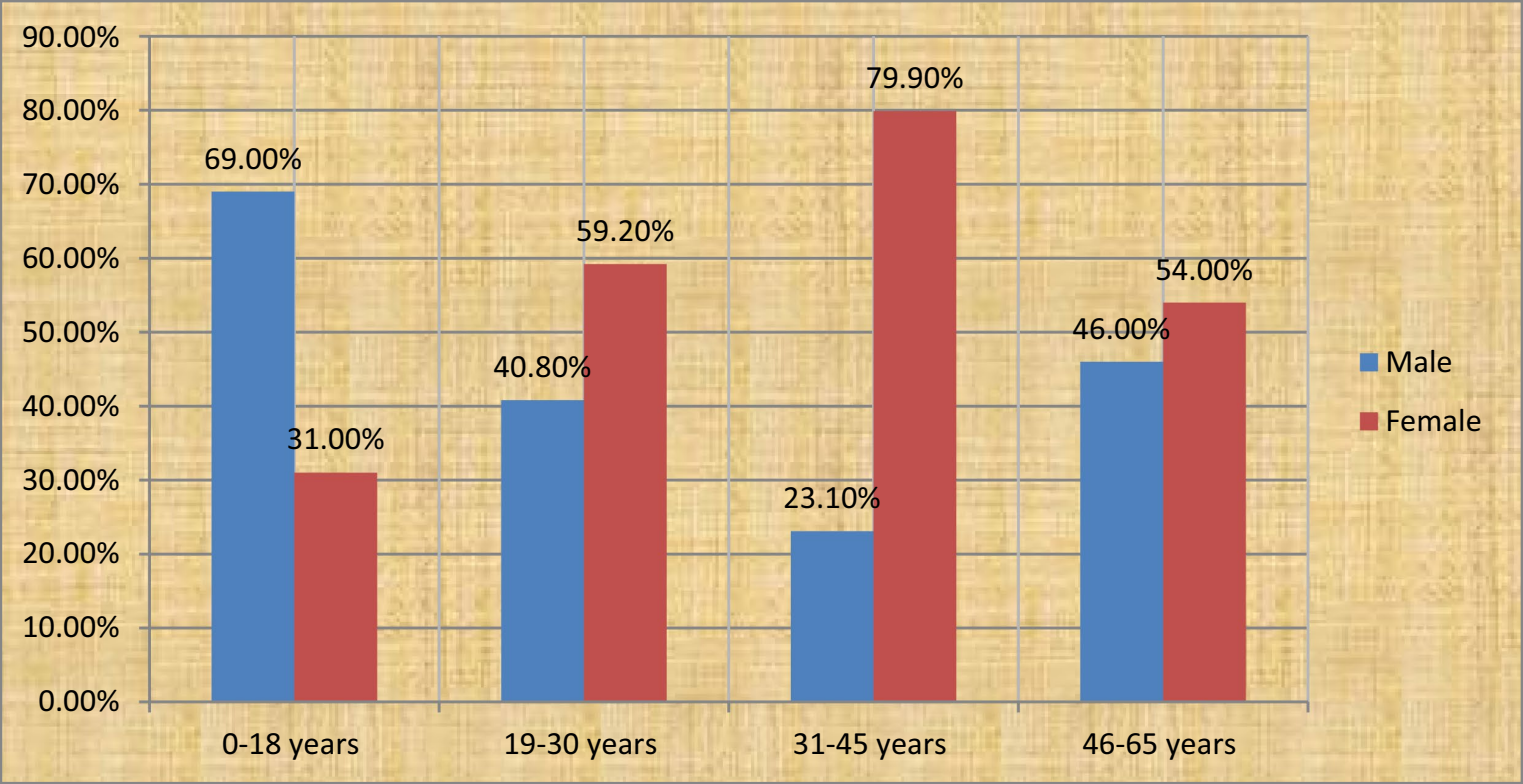
The above bar chart shows the age and sex of patients with cancer at the oncology center of Mekele Comprehensive and Specialized Hospital in 2022.

Data were collected from 354 patients who had all types of cancer before and after phase IV chemotherapy between September 2018 and September 2022. The number of female and male patients was 215 (60.7%) and 139 (39.3%), respectively. The mean age of the respondents was 41.26 (±16.67) years (Figure 1).

### 3.2 Types of cancer in the study participants

**Figure 2:**
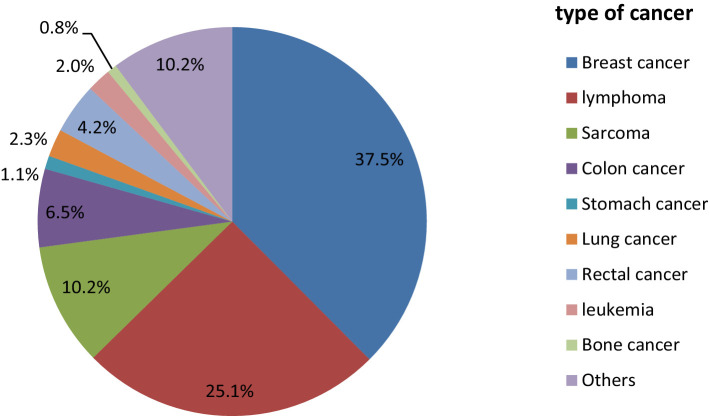
The above pie chart demonstrates the types of cancer among the cancer patients at the oncology center of Mekele Comprehensive and Specialized Hospital in 2022. Others refer to cervical cancer, testicular cancer, uterine cancer, pancreatic cancer, and skin cancer.

Among the total patients, the most common types of cancer were breast cancer (133, 37.6%), lymphoma (89, 25.1%), and sarcoma (36, 10.2%) (Table 1). At the time of first contact, nearly all of them were diagnosed with stage III and stage IV cancer, with 167 (47%) and 159 (45%) cases, respectively (Figure 2).

**Table 1:** Difference in the counts of blood cell types before and after chemotherapy by cancer type among patients with cancer at the oncology center of Mekele Comprehensive and Specialized Hospital in 2022.

Type of cancer	Measure of blood cells pre-and post-chemotherapy	Mean	Mean paired difference	95% CI of mean differences		*t*-value	Df	*p*-value
				Lower	Upper			
Breast cancer	Red blood cell pre-chemotherapy	5.32	0.92	−0.12	1.97	1.74	132	0.040*
	Red blood cell post-chemotherapy	4.39						
	White blood cell pre-chemotherapy	6.46	1.09	0.35	1.84	2.91	132	0.002*
	White blood cell post-chemotherapy	5.36						
	Platelet pre-chemotherapy	332.9	22.7	1.75	43.66	2.14	132	0.017*
	Platelet post-chemotherapy	310.2						
Colon cancer	White blood cell pre-chemotherapy	5.87	0.73	−0.11	1.57	1.79	22	0.042*
	White blood cell post-chemotherapy	5.14						
	Red blood cell pre-chemotherapy	4.70	0.12	0.12	0.56	1.0	22	0.160
	Red blood cell post-chemotherapy	4.59						
	Platelet pre-chemotherapy	320.78	79.56	29.12	129.93	3.27	22	0.002*
	Platelet post-chemotherapy	241.21						
Leukemia	White blood cell pre-chemotherapy	32.82	18.17	−3.23	39.58	2.07	6	0.041*
	White blood cell post-chemotherapy	14.65						
	Red blood cell pre-chemotherapy	2.35	−0.36	−1.38	0.66	−0.86	6	0.790
	Red blood cell post-chemotherapy	2.71						
	Platelet pre-chemotherapy	57.77	−110	−196.4	−24.30	−3.13	6	0.989
	Platelet post-chemotherapy	168.14						
Lung cancer	White blood cell pre-chemotherapy	8.53	0.85	−2.26	3.98	0.64	7	0.268
	White blood cell post-chemotherapy	7.68						
	Red blood cell pre-chemotherapy	5.27	0.48	0.07	0.89	2.74	7	0.014*
	Red blood cell post-chemotherapy	4.79						
	Platelet pre-chemotherapy	296.50	−59.75	−115.36	−4.13	−2.54	7	0.980
	Platelet post-chemotherapy	356.25						
Lymphoma	White blood cell pre-chemotherapy	21.30	6.02	−1.39	13.44	1.61	88	0.05*
	White blood cell post-chemotherapy	15.27						
	Red blood cell pre-chemotherapy	4.37	0.23	0.07	0.38	2.85	88	0.002*
	Red blood cell post-chemotherapy	4.14						
	Platelet pre-chemotherapy	317.00	26.53	−4.44	57.53	1.70	88	0.064
	Platelet post-chemotherapy	290.46						
Others*	White blood cell pre-chemotherapy	9.60	0.27	−2.93	3.48	0.17	35	0.431
	White blood cell post-chemotherapy	9.33						
	Red blood cell pre-chemotherapy	4.56	0.54	0.25	0.82	3.85	35	0.000*
	Red blood cell post-chemotherapy	4.02						
	Platelet pre-chemotherapy	297.69	13.94	−41.74	69.63	0.50	35	0.307
	Platelet post-chemotherapy	283.75						
Rectal cancer	White blood cell pre-chemotherapy	5.39	−0.72	−1.76	0.31	−1.50	14	0.922
	White blood cell post-chemotherapy	6.12						
	Red blood cell pre-chemotherapy	4.47	−0.23	−0.45	−0.01	−2.26	14	0.980
	Red blood cell post-chemotherapy	4.70						
	Platelet pre-chemotherapy	334.40	37.26	−27.74	102.27	1.22	14	0.119
	Platelet post-chemotherapy	297.13						
Sarcoma	White blood cell pre-chemotherapy	6.72	1.23	0.11	2.34	2.44	35	0.016*
	White blood cell post-chemotherapy	5.49						
	Red blood cell pre-chemotherapy	4.66	0.30	−0.00	0.60	2.02	35	0.025*
	Red blood cell post-chemotherapy	4.36						
	Platelet pre-chemotherapy	5.01	0.82	−22.71	59.98	0.91	35	0.183
	Platelet post-chemotherapy	4.19						
Stomach cancer	White blood cell pre-chemotherapy	5.01	0.81	−2.69	4.33	0.74	3	0.256
	White blood cell post-chemotherapy	4.19						
	Red blood cell pre-chemotherapy	3.99	0.43	−1.58	2.45	0.68	3	0.270
	Red blood cell post-chemotherapy	3.56						
	Platelet pre-chemotherapy	348.5	71.5	−141.44	284.44	1.06	3	0.181
	Platelet post-chemotherapy	277						
Bone cancer	White blood cell pre-chemotherapy	5.27	1.63	−4.33	7.59	1.17	2	0.180
	White blood cell post-chemotherapy	3.64						
	Red blood cell pre-chemotherapy	19.34	16.17	−44.54	76.89	1.14	2	0.185
	Red blood cell post-chemotherapy	3.17						
	Platelet pre-chemotherapy	241	115.33	−58.84	289.51	2.84	2	0.052
	Platelet post-chemotherapy	125.66						

Red blood cells and white blood cells were measured in units of 10^6^ cells/mm^3^, while platelets were measured in units of 10^3^ cells/mm^3^. A *p*-value marked * indicates the presence of significant paired differences.

### 3.3 Hematological abnormalities among patients with different types of cancer under chemotherapy

The prevalence of anemia among cancer patients before and after chemotherapy was 25.14% (95%:20.88, 29.94) and 35.54% (95%: 30.75; 40.73), respectively. The frequency of neutropenia among cancer patients was 22.32% (95% 18.26; 26.96) and 27.97% (95%:23.52; 32.88) before and after chemotherapy, respectively. The prevalence of lymphopenia among the study participants was 16.38% (95%:12.87; 20.62) and 17.51% (95% 13.88; 21.84) before and after chemotherapy, respectively (Table 2).

**Table 2:** Hematological parameters of cancer patients before and after chemotherapy at Mekele Comprehensive and Specialized Hospital in 2022.

Hematological parameters	Categories	Frequency before chemotherapy		Frequency after chemotherapy		Cutoff points	X^2^-test	*p*-value
		*N* (%)	[95% CI]	*N* (%)	[95% CI]			
Hemoglobin (female)	Low	51 (23.72%)	18.48; 29.90	72 (33.49%)	27.46; 40.09	<12 g/dL	85.04	0.000
	Normal	157 (73.02%)	66.66; 78.56	142 (66.05%)	59.42: 72.09	12-16 g/dL		
	High	7 (3.25%)	1.55; 6.69	1 (0.47%)	0.064; 03.26	>16 g/dL		
Hemoglobin (male)	low	56 (40.29%)	32.40; 48.71	76 (54.68%)	46.27; 62.81	<13 g/dL	18.79	0.001
	Normal	81 (58.27)	49.84; 66.24	44.60 (44.60)	36.49; 53.01	13-18 g/dL		
	high	2 (1.44)	0.35; 5.63	1 (0.72)	0.09; 5.00	>18 g/dL		
Hematocrit	Normal	225 (63.56)	58.39; 68.42	208 (58.76)	53.53; 63.79	36–56%	68.74	0.000
	Abnormal	129 (36.44%)	31.57; 41.60	146 (41.24)	36.20; 46.46	<36 and > 56%		
Neutrophils	Low	79 (22.32%)	18.26; 26.96	99 (27.97%)	23.52; 32.88	<40%	47.29	0.000
	Normal	137 (38.70%)	33.74; 43.89	136 (38.41%)	33.47; 43.61	40–60%		
	High	138 (38.98%)	34.02; 44.18	134 (37.85%)	28.86; 38.71	>60%		
Lymphocytes	Low	58 (16.38%)	12.87; 20.62	62 (17.51%)	13.88; 21.84	<20%	26.59	0.000
	Normal	185 (52.26%)	47.03; 57.43	189 (53.38%)	48.43; 58.82	20–40%		
	High	111 (31.36%)	26.72; 36.39	103 (29.09%)	24.31; 33.76	>40%		
White blood cells	Low	53 (14.97%)	11.61; 19.09	103 (29.10%)	24.58; 34.06	<4,000	116.70	0.000
	Normal	253 (71.47%)	66.52; 75.94	216 (61.02%)	55.81; 65.97	4,000–11,000		
	High	48 (13.56%)	10.36; 17.55	35 (9.89%)	7.17; 13.47	>11,000		
Platelets	Low	32 (9.04%)	6.45; 12.51	41 (11.58%)	8.63; 15.36	<150,000	66.76	0.000
	Normal	273 (77.12%)	72.43; 81.21	292 (82.48%)	76.93; 85.09	150,000-450,000		
	High	49 (13.84%)	10.61; 17.86	21 (5.93%)	3.89; 8.93	>450,000		

White blood cells and platelets were measured by cells/μL.

### 3.4 Difference in the count of blood cell types pre-and post-chemotherapy among patients with different types of cancer

In patients with breast cancer, all types of blood cell counts showed a significant decrease after chemotherapy. White blood cells and red blood cells were reduced after chemotherapy among sarcoma and lymphoma patients, with paired mean differences of 6.02 × 10^6^ cells/mm^3^, 0.23 × 10^6^ cells/mm^3^, 1.23 × 10^6^ cells/mm^3^, and 0.30 × 10^6^ cells/mm^3^ (Table 1).

In patients with stomach, rectal, and bone cancers, there was no significant difference in the counts of all blood cell types before and after chemotherapy. This study also revealed that there was an increase in platelets and red blood cells after chemotherapy among leukemia patients, although this increase was not significantly different.

The “Others*” category refers to cervical cancer, testicular cancer, uterine cancer, pancreatic cancer, and skin cancer.

### 3.5 Difference in the count of blood cell types pre-and post-chemotherapy among all types of cancer patients

The profile of blood cell types before and after chemotherapy for all types of cancer is shown in Table 3. Three types of blood cells—red blood cells, white blood cells, and platelets—were measured at the time of diagnosis and after phase IV chemotherapy. The mean values of red blood cells and white blood cells were measured in cells per mm^3^ × 10^6^. The mean value of platelets was measured in cell mm^3^ × 10^3^ (Table 1).

**Table 3:** Comparison of the levels of blood cell types between the pre-and post-chemotherapy periods at Mekele Comprehensive and Specialized Hospital in 2022.

Pairs	Measure of blood cells pre-and post-Chemotherapy	Mean	Mean paired difference	95% CI of mean differences		*t*-value	Df	*p*-value
				Lower	Upper			
Pair1	Red blood cell pre-chemotherapy	4.90	0.63	0.17	1.10	2.67	353	0.008
	Red blood cell post-chemotherapy	4.27						
Pair2	White blood cell pre-chemotherapy	10.99	2.49	0.55	4.44	2.52	353	0.012
	White blood cell post-chemotherapy	8.50						
Pair3	Platelet pre-chemotherapy	316.82	23.51	9.57	37.47	3.31	353	0.001
	Platelet post-chemotherapy	293.30						

As shown in Table 3, all profiles of blood cell types showed a decrease post-chemotherapy compared to pre-chemotherapy. The mean decrease in platelets was 23.51 × 10^3^ cells/mm^3^ (*p* = 0.001), in red blood cells was 0.63 × 10^6^ cells/mm^3^ (*p* = 0.08), and in white blood cells was 2.49 × 10^6^ cells/mm^3^ (*p* = 0.012).

## 4 Discussion

Chemotherapy is a treatment with high toxicity to normal cells. Different cancer patients who receive chemotherapy often experience alterations in hematological profiles. Cancer chemotherapy treatments have devastating effects on the patient’s hematopoietic system (16).

Cancer treatment has adverse effects on normal blood cell types. The drugs used in this treatment are cytotoxic to active cells (9, 20, 21). Cancer cells are abnormal and can multiply uncontrollably, while the disadvantage of chemotherapy drugs occurs when these drugs disrupt the normal function and multiplication of healthy cells (1). Cancer patients who undergo chemotherapy face substantially life-threatening complications, such as low hemoglobin levels and a reduction in white blood cells. This leads to anemia and increased susceptibility to various infections (16).

In this retrospective study, the paired mean values of blood cell types were measured pre-and post-chemotherapy in the patients. The result of this study revealed that the mean count of red blood cells was reduced post-chemotherapy compared to pre-chemotherapy in the patients with breast cancer, lymphoma, lung cancer, and sarcoma. This study is in line with a study conducted on the effect of chemotherapy and radiotherapy on red blood cells and hemoglobin in cancer patients (9, 22). This suppression of red blood cells might be because chemotherapy suppresses the bone marrow, which produces normal and active red blood cells (5, 23). Another explanation for this reduction in red blood cells is that many drugs that are used to treat cancerous cells damage normal red blood cells. Therefore, the reduction in red blood cells leads to a decrease in hemoglobin and hematocrit. Erythropoiesis production may be compromised because of chemotherapy, and this disruption leads to a reduction in red blood cells. Drugs of chemotherapy also induce renal toxicity. Nephrotoxic drugs used in chemotherapy significantly disrupt erythropoietin production. Another reason for this reduction may be a decrease in erythropoietin levels. Chemotherapy has a fragility effect on red blood cells (22). In this study, the prevalence of anemia before and after chemotherapy was 25.14 and 35.59%, respectively. This study is similar to a study conducted in Gondar (24). This similarity may be due to the use of similar chemotherapy cycles to treat patients with cancer in both studies. Another reason for this similarity may be that the regimen of chemotherapy used to treat patients in both study areas is similar. However, the prevalence of anemia among the study participants in the current study was lower than that in a study conducted in northwest Ethiopia (25). This difference might be due to the different chemotherapy regimens used to treat patients. Another reason may be that the number of treatment cycles in the current study is lower compared to the previous study. High numbers of chemotherapy cycles are associated with chemotherapy-induced anemia.

The platelet count in this study showed a significant difference between pre-and post-chemotherapy levels in the patients with cancer. This decrease in platelet counts post-chemotherapy might be due to the destruction of megakaryocyte progenitors during the early stage of differentiation caused by chemotherapy. The difference may be due to the disruption of hormones produced in the kidney and liver that are involved in platelet production by chemotherapy (2, 3). The platelet count post-chemotherapy compared to pre-chemotherapy was low. This study contrasts with a meta-analysis conducted on ovarian cancer (19). This difference may be due to the fact that this study included all types of cancer, while the mentioned study was focused only on ovarian cancer. The results of this study are similar to those of a study conducted in Ethiopia comparing biochemical and hematological profiles, as well as another study on drug-induced neutropenia (26, 27). This similarity may be due to the similar characteristics of the study participants in terms of social, economic, and environmental factors.

According to the result of this study, the white blood cell count decreased after chemotherapy compared to pre-chemotherapy among the patients with breast cancer, colon cancer, leukemia, lymphoma, and sarcoma. This study is consistent with a study conducted on responses to chemotherapy and remission induction therapies (28, 29). This similarity may have occurred because the administered drugs for chemotherapy were similar. Another reason for this consistent result may be that chemotherapy has a cytotoxic effect that disrupts the normal production of white blood cells. In contrast, other studies have revealed a significant increase in white blood cells post-chemotherapy compared to pre-chemotherapy (30). This increase may be due to chemotherapy inducing infections in these patients, which leads to an increase in white blood cells.

In this study, the types of cancer and their frequencies were identified. Breast cancer, lymphoma, and sarcoma were the top three cancer types in this study. This distribution is consistent with trends in global cancer epidemiology, where breast cancer continues to rank among the most commonly diagnosed cancers, especially in women (31). The substantial burden of hematologic and soft tissue cancers in our sample was further suggested by the high frequency of lymphoma and sarcoma. This finding is similar to those of studies conducted in Logos, Nigeria, and Ethiopia (23, 32). This similarity may have occurred because the socio-demographic and biological characteristics of the study participants are similar. Another reason for this may be that they have similar risk factors for these cancer types. The late-stage manifestation of cancer at the time of initial interaction is one of our study’s most alarming findings. There was a significant delay in diagnosis and treatment initiation, as nearly all patients were diagnosed at stages III (47%) and IV (45%). Many factors could be responsible for this delay, such as restricted access to healthcare services, ignorance of early symptoms, and even socioeconomic constraints that impede prompt medical evaluation. Lymphoma was the second most prevalent type of cancer in our study. This study is similar to a study conducted at Ayder Hospital, Ethiopia (27). The consistency of this finding may be due to similarities in socio-demographic characteristics. Another reason for this similarity may be that there are similar determinants for lymphoma cancer. Infections, weakening of the immune system, and exposure to chemicals are risk factors for lymphoma. The study participants in the current study and the other study may have had similar exposure status.

The findings of this study revealed a difference in the measurement of hematological parameters. The level of hemoglobin post-chemotherapy decreased compared to pre-chemotherapy. This observed difference may be related to adverse reactions in patients with normal baseline levels of hemoglobin (33, 34). Chemotherapy-induced bone marrow damage is accompanied by nerve injury to the bone marrow. Another reason for the observed difference might be related to dysfunction in hematopoietic production. This results in chronic bone marrow damage caused by chemotherapy (35, 36).

Lymphocytes, a type of white blood cell involved in immune responses, are specifically sensitive to chemotherapy and often decrease as a result. Their depletion, known as lymphopenia, weakens the adaptive immune system, increasing the patient’s vulnerability to viral infections and reducing overall immune surveillance against malignancies. The current study is similar to other studies that have shown that chemotherapy-induced lymphopenia is associated with poorer overall survival in cancer patients (37, 38).

Neutrophil is a vital component that plays a significant role in fighting microorganisms. In the current study, there was a decrease in the levels of neutrophils pre-chemotherapy compared to post-chemotherapy. This observed difference is in line with previous studies (27, 39). This similarity may be related to the same chemotherapy regimen given to these patients. Another reason for this similarity may be the neutrophil-suppressive effects of chemotherapy in the present study.

In the present study, leukopenia was observed after chemotherapy. However, this finding contrasts with the results of a study on leukocytosis, thrombocytosis, and early mortality among cancer patients initiating chemotherapy (40). This contrasting finding may be due to tissue damage, inflammation, and emotional stress after chemotherapy, which can lead to increased leukocyte production, as observed in the results of the previous study.

### 4.1 Strengths and limitations of the study

- Strength: All types of cancer patients were included in the study

### 4.2 Limitations

- Since data were collected from a secondary source, some data were not registered
- Other biochemical profiles were not considered
- Changes from phase to phase were not compared

## 5 Conclusion and recommendations

Chemotherapy has a reducing effect on lymphocytes, neutrophils, WBCs, hemoglobin, and platelets. These reductions not only compromise immune function but also increase the risk of infections, bleeding disorders, and treatment delays. Comparative studies have also shown that severe lymphopenia and neutropenia can significantly impact patient prognosis, leading to higher morbidity and mortality if left unmanaged. We also recommend that clinicians implement proactive monitoring and personalized management strategies to balance effective chemotherapy treatment while minimizing hematological toxicity.

## Data Availability

The raw data supporting the conclusions of this article will be made available by the authors, without undue reservation.
